# Effect of Nanoparticle Size and Concentration on Pool Boiling Heat Transfer with TiO_2_ Nanofluids on Laser-Textured Copper Surfaces

**DOI:** 10.3390/nano12152611

**Published:** 2022-07-29

**Authors:** Armin Hadžić, Matic Može, Klara Arhar, Matevž Zupančič, Iztok Golobič

**Affiliations:** Faculty of Mechanical Engineering, University of Ljubljana, Aškerčeva 6, 1000 Ljubljana, Slovenia; armin.hadzic@fs.uni-lj.si (A.H.); klara.arhar@fs.uni-lj.si (K.A.); matevz.zupancic@fs.uni-lj.si (M.Z.); iztok.golobic@fs.uni-lj.si (I.G.)

**Keywords:** nanoparticles, nanofluids, boiling, heat transfer, heat transfer enhancement, nanoparticle deposition

## Abstract

The enhancement of boiling heat transfer has been extensively shown to be achievable through surface texturing or fluid property modification, yet few studies have investigated the possibility of coupling both enhancement approaches. The present work focuses on exploring the possibility of concomitant enhancement of pool boiling heat transfer by using TiO_2_-water nanofluid in combination with laser-textured copper surfaces. Two mass concentrations of 0.001 wt.% and 0.1 wt.% are used, along with two nanoparticle sizes of 4–8 nm and 490 nm. Nanofluids are prepared using sonification and degassed distilled water, while the boiling experiments are performed at atmospheric pressure. The results demonstrate that the heat transfer coefficient (HTC) using nanofluids is deteriorated compared to using pure water on the reference and laser-textured surface. However, the critical heat flux (CHF) is significantly improved at 0.1 wt.% nanoparticle concentration. The buildup of a highly wettable TiO_2_ layer on the surface is identified as the main reason for the observed performance. Multiple subsequent boiling experiments using nanofluids on the same surface exhibited a notable shift in boiling curves and their instability at higher concentrations, which is attributable to growth of the nanoparticle layer on the surface. Overall, the combination of nanofluids boiling on a laser-textured surface proved to enhance the CHF after prolonged exposure to highly concentrated nanofluid, while the HTC was universally and significantly decreased in all cases.

## 1. Introduction

Pool boiling heat transfer has been widely used in numerous engineering systems, such as aircraft and spacecraft thermal management, high power electronics cooling, heat exchangers, nuclear reactors, air conditioning, thermal power generation, etc. [1,2,3,4,5,6]. Compared to natural and forced convection without phase change, the advantage of pool boiling is a higher heat removal rate from a surface while maintaining a low superheat (i.e., the temperature difference between the surface and the boiling liquid), which is an important advantage for its use in dissipating highly concentrated thermal loads.

Pool boiling is the process of vaporization at the solid–liquid interface. and it occurs when the temperature of the surface exceeds the saturation temperature of the liquid at the given pressure. The characteristics of the pool boiling heat transfer process can be described by the boiling curve, which was first reported by Nukiyama [7]. In the first phase of boiling heat transfer, all of the heat is dissipated through single-phase natural convection. When the surface superheat is high enough, vapor bubbles begin to form in the cavities on the surface (i.e., heterogeneous boiling takes place), which represents the onset of nucleate boiling (ONB) and the inception of the nucleate boiling phase. The nucleate boiling heat transfer regime is the most effective heat transfer region of the pool boiling process due to very high heat transfer coefficients at low surface superheat values, making it the most suitable method for various engineering applications. The heat transfer coefficient (HTC), representing the ratio between dissipated heat flux and the corresponding wall superheat, is the most common metric to describe the heat transfer intensity. With increasing heat flux, the number of active nucleation sites on the surface increases. When the population of bubbles becomes too high (at a high heat flux), neighboring bubbles coalesce extensively (i.e., merge on or above the surface), forming an insulating blanket of vapor covering the heating surface and thereby significantly decreasing the heat transfer intensity. This phenomenon is known as the boiling crisis, and the maximum heat flux associated with its incipience is the critical heat flux (CHF). After CHF onset, the boiling process transitions towards film boiling, which is characterized by a high increase in wall temperature and a large decrease in the HTC [8,9].

While boiling heat transfer represents an efficient cooling method, the HTC and CHF need to be enhanced to meet the specifications of certain applications, allowing for the safe and efficient operation of such systems. The enhancement of heat transfer in the nucleate boiling regime can be achieved in various ways, including by (i) changing the characteristics of the boiling surface, (ii) modifying the surface-fluid interaction, (iii) modifying the working fluid, or (iv) changing the operating conditions [10,11]. The aim of these methods is generally to lower the ONB and increase both the CHF and the HTC [11,12,13]. Most approaches to boiling enhancement only consider one technique, meaning that possible combinations and synergies between more techniques are largely unexplored. This is addressed in the present study, where surface modification via laser texturing is coupled with fluid modification through the addition of nanoparticles, with the aim of obtaining superior boiling performance to that using a single enhancement approach.

### 1.1. Methods for Intensifying Boiling Heat Transfer

In recent years, researchers have thoroughly studied different techniques for intensifying boiling heat transfer, such as adding nanoparticles to the base fluid or modifying the surface characteristics of the boiling surface, namely its roughness, porosity, wettability, wickability, etc. [13,14,15,16,17,18]. Surface modification may be performed on the macro-, micro- or nanoscale [11]. The techniques for the modification of surface morphology can generally be divided into physical and chemical categories [19]. Common physical techniques include electro-deposition [20] and pulsed laser deposition [21], while in most studies, the chemical techniques are chemical vapor deposition [22], the sol-gel technique, and electrochemical deposition [23]. Overall, surface modification aims to alter the surface wettability and microstructure, which have a profound impact on the boiling process and the associated heat transfer parameters [16,24,25]. Free surface energy, roughness of the surface, and its micro- and nanotexture have a strong influence on wettability [10,26,27]. Based on the contact angle of a liquid droplet on the surface, the latter can be classified as phobic or philic to the liquid. As water is the most common testing medium, the surfaces are typically classified as hydrophilic (contact angle < 90°) or hydrophobic (contact angle > 90°). Based on previous experimental findings, the ONB will occur at a lower superheat, and CHF will be decreased on hydrophobic surfaces, while hydrophilic surfaces will exhibit delayed ONB, but increased CHF [10,28,29].

### 1.2. Boiling of Nanofluids

A common method of modify the properties of the coolant in boiling applications is the addition of various types of nanoparticles to the base fluid [30,31,32]. This changes its thermophysical properties, but more importantly, the nanoparticles are deposited onto the boiling surface during boiling, creating micro- and nanostructures that can favorably affect the boiling process [33]. The available literature mostly suggests that the boiling of nanofluids leads to an improvement of the HTC and the CHF, while some few studies make the opposite conclusion [34]. The increase in HTC and CHF tends to be correlated with increasing the concentration of nanoparticles, up to a certain point. At excessive concentrations, the HTC begins to decrease, while the CHF will remain unaltered [35]. At high nanoparticle concentration, the deposited layer on the surface becomes thicker, which leads to greater thermal resistance and decreases the HTC [35]. Due to the deposition of nanoparticles on the surface and their large effect on heat transfer during boiling, Fang et al. [31] suggested that further experiments on nanofluid boiling should be combined with a comprehensive study of the influence of nanoparticle deposition time for different concentration ranges. Long-term experiments on the variation of nanoparticle deposition time should be performed, and correlations to predict the evolution of deposited nanoparticle layers should be proposed. In addition to the concentration, the material and size of the nanoparticles and the preparation of the nanofluid also affect the boiling performance. Dadhich et al. [36] and Fang et al. [31] pointed out that there is a significant need for a database of thermal properties for different materials in different size ranges of nanoparticles.

The deposition of nanoparticles from the nanofluid during boiling is the most common enhancement strategy involving modified fluids. Manetti et al. [37] conducted an experimental study of HTC in pool boiling of deionized water and Al_2_O_3_-water nanofluid with low (0.0007 vol.%) and high (0.007 vol.%) volume concentration on smooth and rough copper surfaces within a heat flux range of 100 to 800 kW m^−2^. They observed that HTC increased at low concentrations at average heat flux values, which was related to increasing the radius of the cavities through deposition of nanoparticles on the surfaces due to boiling. Increasing the heat flux led to a decrease in the HTC caused by the increased deposition rate of nanoparticles on the rough surface and the filling of the cavities with nanoparticles. Ahmed et al. [38] investigated pool boiling heat transfer performance on horizontal flat copper surfaces using nanofluid and pure water. They used 40–50 nm alumina nanoparticles to prepare three different nanoparticle concentrations (0.01 vol.%, 0.1 vol.%, and 1 vol.%). After performing boiling experiments, the nanoparticle-coated surfaces were used for pool boiling experiments using pure water. During the nanofluid boiling experiments, the authors concluded that the concentration of nanoparticles has a major effect on the heat transfer performance. At lower concentrations, the rate of deposition of the particles is lower, resulting in a greater enhancement of heat transfer, which can be attributed to the fact that the increased thermal conductivity of nanofluids has a greater dominance than the effect of nanoparticle deposition on the surface. On the other hand, the boiling of pure water on the surface coated with nanoparticles showed that high deposition rates lead to an improvement in heat transfer, which was explained by a less uniform deposition layer on the surface. Huang et al. [39] studied the enhancement of boiling on nickel wires coated with TiO_2_ nanoparticles in pure water. The coating was produced by electrical heating of the wire in nanofluids with concentrations from 0.01 to 0.1 wt.% and heat flux up to 1000 kW m^−2^. Experimental results of pure water boiling showed an enhancement of the CHF of up to 82.7% for coated nickel wire prepared in 0.1 wt.% nanofluids. On all nickel-coated wires, the HTC deteriorated due to the higher thermal resistance caused by the deposition of nanoparticles. Kiyomura et al. [40] investigated boiling heat transfer performance of surfaces coated with Fe_2_O_3_ nanoparticles in Fe_2_O_3_ water-based nanofluid at a high (0.29 g/L) and a low concentrations (0.029 g/L). The results showed the highest HTC values on coated copper surfaces with low mass concentration, and with increasing the concentration, the roughness of the surfaces increased. Salimpour et al. [41] performed boiling experiments on smooth and rough copper surfaces using iron-oxide-water-based nanofluid at low and high heat fluxes. They found that at low heat fluxes on smooth surfaces, and at high heat fluxes on rough surfaces, the deposition of nanoparticles on the surface enhanced the heat transfer during boiling. According to other research conducted in this field, there is no clear understanding of how the boiling heat transfer performance is changed by the various effects of nanoparticle deposition during nanofluid boiling [42,43].

In addition to in situ nanoparticle deposition, dip coating and drop casting techniques were also proposed for boiling applications. An example is the study by Yim et al. [44], who investigated the surface wettability in nucleate pool boiling on aluminum surfaces coated with TiO_2_ nanoparticles from 1 wt.% TiO_2_ ethanol-based nanofluids, using a drop casting technique. The obtained results showed that the performance of nucleate pool boiling was 64.1% higher for TiO_2_-coated surfaces than for the bare surfaces.

### 1.3. Boiling on Laser-Texured Surfaces

Laser texturing is a very effective method for locally or globally changing the morphology of the surface to intensify boiling heat transfer. Može et al. [45] demonstrated a strong enhancement of boiling performance of water on superhydrophobic and superhydrophilic surfaces textured with a nanosecond fiber laser. By combining laser texturing with a superhydrophobic coating, they showed significant improvement in HTC and found that the intensification of boiling heat transfer is possible with a suitable surface morphology where the Wenzel wetting regime is achieved. Kurse et al. [46] studied the pool boiling heat transfer of deionized water on laser-textured surfaces. Microstructures were fabricated on stainless steel using a femtosecond laser. It was found that CHF and the maximum heat transfer coefficient on laser-textured surfaces were improved compared to those on the polished reference surface. It was also found that the improvement of CHF is related to the wetting and wicking capability of the surface, which allows for the replenishing of the evaporating liquid and affects the delay of the CHF. The study of the nucleate pool boiling of water and water-ethanol mixtures on untreated and laser textured stainless steel foils showed a significant improvement in boiling performance on laser-textured surfaces compared to the untreated surfaces for pure liquids and binary mixtures. The improvement in boiling performance is directly related to the microcavities, which act as active nucleation sites [47]. Serdyukov et al. [48] investigated nucleate pool boiling of water on laser textured silicon surfaces. Their results showed an enhancement of the heat transfer coefficient by up to 49.5%, compared to a rough silicon sample, and by up to 234%, compared to a polished sample. Additionally, the study showed that laser texturing of the surfaces resulted in a remarkable increase in the frequency and density of nucleation sites. It was concluded that a decrease in the diameter of the departure bubble and a lower nucleation temperature are characteristics that are associated with the laser-modified silicon surfaces. The study of the stability of copper surfaces before and after functionalization by laser texturing performed with a nanosecond fiber laser was presented by Može et al. [49]. The study showed the enhancement of CHF by up to 90% and HTC by up to 115% on textured surfaces compared to the reference surface. The results also demonstrate the tendency of constant shifts of boiling curves in each experimental run, while the shifts on laser textured surfaces did not occur after the second run. This fact is confirmed by changes in the morphological and chemical structure of the surface after the first onset of CHF and is associated with the effects of low-temperature annealing. Finally, only one study, published by Karthikeyan et al. [50], was found to have previously combined surface laser texturing and the use of nanofluids to enhance boiling heat transfer. The latter authors reported a notable enhancement of boiling heat transfer, but the nanofluid used was rather unconventional (carbon nanotubes dispersed in ethanol or water).

### 1.4. Scope and Aim of this Study

There are few studies that investigate the boiling performance of nanofluids on pre-modified surface (e.g., laser-textured surfaces) [51,52], despite the seemingly great potential for concomitant and synergistic enhancement of boiling performance. To fill this knowledge gap, we investigated the pool boiling heat transfer performance of TiO_2_-water nanofluids prepared with two mass concentrations (0.001 and 0.1 wt.%) and with two different sizes of nanoparticles (small: 4–8, and large: 490 nm), on laser textured copper surfaces. Five consecutive measurements were performed on each laser-textured surface under pool boiling conditions. The surface morphology of deposited nanoparticles was analyzed using scanning electron microscopy (SEM), and the wettability changes were recorded through water contact angle (WCA) measurements. The results were analyzed through multiple comparisons to elucidate the effect of nanoparticle size and concentration on possible additional enhancement or deterioration of boiling performance of the laser-textured copper surfaces.

## 2. Methods

### 2.1. Sample Preparation and Amalysis

Samples for boiling experiments were prepared on high purity copper (>99.9% Cu). Each sample was first sanded using P1200 and P2000 grit sandpaper to achieve a surface roughness of approx. 0.15 μm. Afterwards, the samples were cleaned using isopropanol and lint-free wipes. One sample was tested without any further treatment, and it is denoted as REF (i.e., untreated reference sample). All other samples underwent direct laser texturing immediately before the boiling experiments.

To perform the laser texturing, a nanosecond pulsed fiber laser was used (FL-mark-C with JPT Opto-electronics Co., Ltd. “M7 30 W” MOPA source, Shenzhen, China). The laser system is equipped with an OPEX F-Theta lens with a focal distance of 100 mm and working field of 70 × 70 mm^2^. A pattern of equidistant parallel lines (Δ*x* = 60 μm) was used to create a channel-like microstructure on each sample. The pulse frequency was set to 110 kHz, the pulse duration to 45 ns, and the full power of 30 W was applied. With the focal beam diameter of ~25 μm and laser beam quality parameter M^2^ ≤ 1.3, the average laser pulse fluence was calculated to be ~56 J cm^−2^.

**Figure 1:**
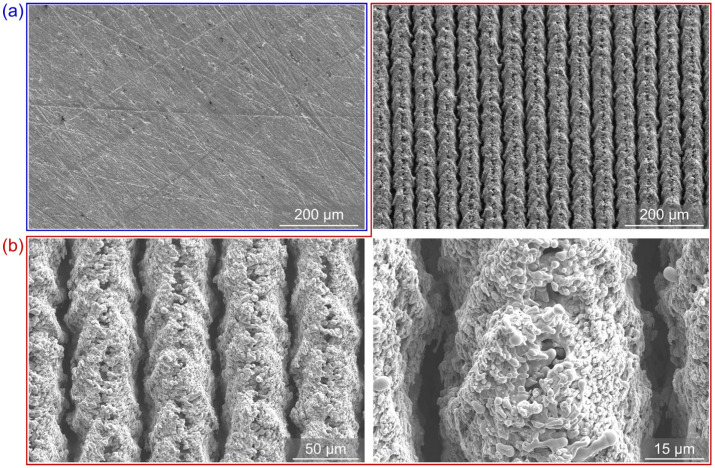
SEM images of the (**a**) untreated reference sample REF (blue frame) and (**b**) laser-textured sample LT (red frame) before exposure to boiling.

The morphology and elemental composition of the samples were analyzed using scanning electron microscopy (ThermoFisher Scientific Quattro S, Waltham, MA, USA) and energy-dispersive X-ray spectroscopy (Oxford Instruments Ultim Max 65, Abingdon, UK). SEM images of the reference sample REF and laser-textured sample (LT) before exposure to boiling are shown in Figure 1.

The analysis of sample wettability was performed using a goniometer to record the static contact angles with water. Cleaning of the samples was performed with a UV/ozone cleaner (Ossila) to remove hydrocarbon contaminants after boiling/storage. Contact angles were measured: (i) before boiling, (ii) immediately after boiling experiments, (iii) 7 days after the boiling process, (iv) after UV/ozone cleaning (7 days after the experiments), and finally (v) three days after UV/ozone cleaning. Measurements were conducted using a goniometer (Ossila, ±1°). Five drops were deposited onto different parts of the surface, and the contact angles were recorded and averaged.

### 2.2. Boiling Performance Evaluation

**Figure 2:**
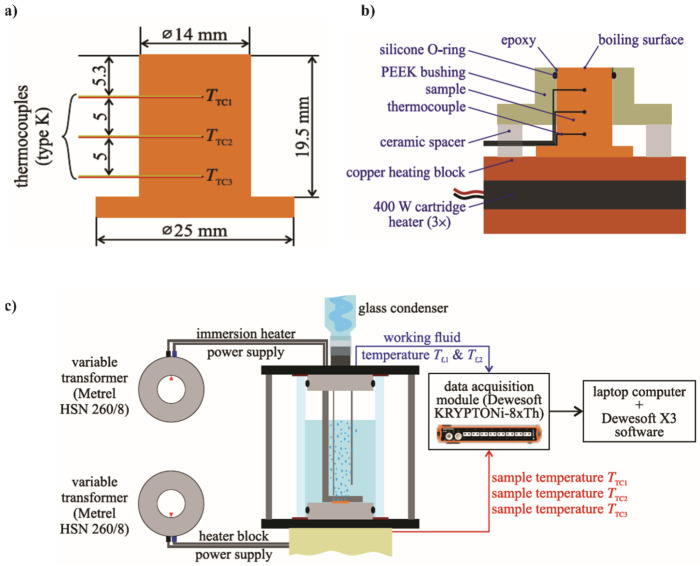
Dimensions of the sample including the position of thermocouples (**a**), cross section of the sample and the heating assembly (**b**), and experimental setup (**c**).

The boiling performance evaluation of nanofluids on laser-textured copper surfaces was performed using a previously developed experimental setup shown in Figure 2 [53]. A glass cylinder with an inner diameter of 60 mm was placed between two stainless steel flanges, thus forming the boiling chamber. The latter was filled with 200 mL of the working fluid during experiments. As shown in Figure 2b, the sample was mounted on a copper heating block, and inserted through the bottom stainless-steel flange into the boiling chamber. PEEK bushing, a ring of flexible epoxy glue, and a silicone O-ring were used to ensure sealing, limit heat loss, and prevent parasitic boiling. Before every experiment, the working fluid was preheated and degassed with an immersion heater, which was controlled by a variable transformer. An immersion heater was also used to maintain the saturated state of the working fluid during the boiling experiment. The cartridge heaters, positioned inside the heating block, were used for the generation of heat and were also controlled by a variable transformer. All measurements were performed at atmospheric pressure, and the stability was confirmed by the stable temperature of the saturation. Vapor produced during measurements went to the water-cooled glass condenser and returned to the boiling chamber.

The temperatures inside the sample were measured by utilizing three K-type thermocouples spaced 5 mm apart from one another. The thermocouple closest to the boiling surface was positioned 5.3 mm from the top of the sample, as shown in Figure 2c. Two further K-type thermocouples were submerged into the boiling chamber at different heights to measure the temperature of the working fluid. A KRYPTONi-8xTH DAQ device was used for collecting all temperature signals as raw voltages. The temperature of each cold junction was recorded internally and used to offset the measurements to obtain correct temperature readings. The calculation of temperatures based on offset voltages was performed, utilizing NIST 9th degree polynomial. Data from the DAQ device were acquired using Dewesoft X3 software at a frequency of 10 Hz.

### 2.3. Data Reduction and Measurement Uncertainty

The calculation of relevant heat transfer parameters is based on measured temperatures. The parameters which are most relevant in boiling heat transfer systems are heat transfer coefficient, heat flux, and superheat of the surface. The methodology used for the calculation of the spatial temperature gradient between the highest positioned and the lowest positioned thermocouple in the sample utilizing the following linear interpolation (1) is suggested in [54]:

(1) $$\frac{∆T}{∆x}=\frac{T_{\mathrm{TC}3}-T_{\mathrm{TC}1}}{2∆x_{1}}$$

Heat losses during the experiments are neglected because of favorable insulative properties achieved using a very low thermal conductivity material (PEEK). Consequently, a simplified case of 1D conduction through the sample towards the boiling surface is considered. The heat flux is calculated using Fourier’s law of conduction:

(2) $$\dot{q}=-k\frac{∆T}{∆x}$$

To accurately calculate the heat flux, thermal conductivity needs to be precisely evaluated. This is achieved by using a temperature-dependent value of thermal conductivity. Based on the average of all three temperatures measured within the sample, the thermal conductivity is calculated at the mean temperature (*T*) of all three temperatures in the sample according to the following equation:

(3) $$k\left(T\right)=0.000283T^{2}-0.1646T+378.07,$$

The laser-flash measurement method of thermal diffusivity at various temperatures, and the determination of thermal conductivity based on temperature-dependent density and specific heat capacity are used to determine the latter expression. All temperature values are expressed in °C, and the thermal conductivity, as the result value, is returned in W m^−1^ K^−1^. The temperature of the boiling surface is calculated by linear extrapolation using the previously determined heat flux and the temperature measured with the highest positioned thermocouple in the sample. The thermal conductivity is first determined at the highest positioned thermocouple in the sample, *T*_TC1_, utilizing Equation (3) to obtain an estimate of surface temperature. The mean temperature of the top part of the sample is determined as the arithmetic average value between the estimated surface temperature and the temperature of the highest positioned thermocouple. The mean temperature of the top part of the sample is used to determine the average thermal conductivity, which is then used to evaluate the surface temperature with increased accuracy. The saturation temperature of the working fluid is calculated as the arithmetic average value of the temperatures measured with two immersed thermocouples. The difference between the saturation temperature of the working fluid and surface temperature is the surface superheat (*T*_w_ − *T_sat_*). Dividing the heat flux by the corresponding surface superheat is used to determine the heat transfer coefficient:

(4) $$h=\frac{\dot{q}}{T_{w}-T_{sat}},$$

### 2.4. Peparation of Nanofluids

The nanofluids used were prepared by dispersing different amounts of nanoparticles in the base fluid (twice-distilled water). The nanofluids used in this study were prepared and stabilized using ultrasonic vibration, without using surfactants or adjusting the pH value. TiO_2_ nanoparticles were chosen for the study because of their favorable chemical and physical stability and their hydrophilicity, which was previously shown to enhance the CHF value during boiling experiments. Two nanoparticle size ranges were used to examine the effect of their size, especially relative to the size of laser-induced surface structures. One type of nanofluid was prepared using very small nanoparticles (sized below 10 nm, much smaller than the majority of structures resulting from laser texturing), while the other type used nanoparticles that were two orders of magnitude larger, with diameters close to 500 nm (close to the scale of laser-induced surface structures). Furthermore, two nanofluids concentrations were tested for each type of nanofluid. Most studies typically report nanofluid concentrations in the range from 0.001 wt.% to 0.1 wt.%. Therefore, we opted to use the two extreme values, again separated by two orders of magnitude, in order to evaluate the effect of nanoparticle concentration on boiling performance.

One type of nanofluid was prepared using small nanoparticles, with a size of 4 to 8 nm (Carl Roth, Karlsruhe, Germany; ROTInanoMETIC ≥ 99.9%), and the second type was prepared using large nanoparticles, with a size of 490 nm (Nanografi Nano Technology, Ankara, 99.995+%). Both types of nanofluids were prepared at two different mass concentrations: 0.1 and 0.001 wt.%. The third type of nanofluid was prepared with 0.05 wt.% of the large-sized and small-sized nanoparticles, respectively. Twice-distilled water was degassed for 45 min via vigorous boiling before it was used to prepare the nanofluids to reduce the amount of entrapped gases and also reduce the need for further degassing in the boiling chamber, where deposition of nanoparticle on the immersion heater reduces the concentration of the nanofluid in an uncontrolled way. Nanofluids were sonicated for 1 h immediately after preparation in an ultrasonic bath (ASonic, Ultrasonic Cleaner-Pro 30, 40 kHz, 120 W). To stabilize the solution before use in the experiment, the nanofluid was again sonicated for 1 h. The performed experiments are summarized in Table 1.

Overall, the used nanoparticle sizes and concentrations are based on the extreme values reported in the literature, and the corresponding extreme combinations, as used here, are expected to allow for the generalization of the results.

### 2.5. Measurement Protocol

After degassing the working fluid, five experimental runs were performed for each combination of the sample and working fluid. During each experimental run, the heat flux was continuously increased at a rate of 2 kW m^−2^ s^−1^. The chosen methodology of a slow continuous increase in the heat flux can be considered as quasi-stationary, as shown by Može et al. [45]. The boiling curve was recorded until the CHF was reached or, when that was not possible, until a surface superheat was reached (~100 K, representing a safety limit of the experimental setup). When an experimental run was finished, cartridge heaters were turned off, and the sample was left to cool down on its own. The duration of each run was approximately 45 min, while all 5 runs were completed in a 5-hour period.

## 3. Results and Discussion

### 3.1. Boiling Heat Transfer with Water

**Figure 3:**
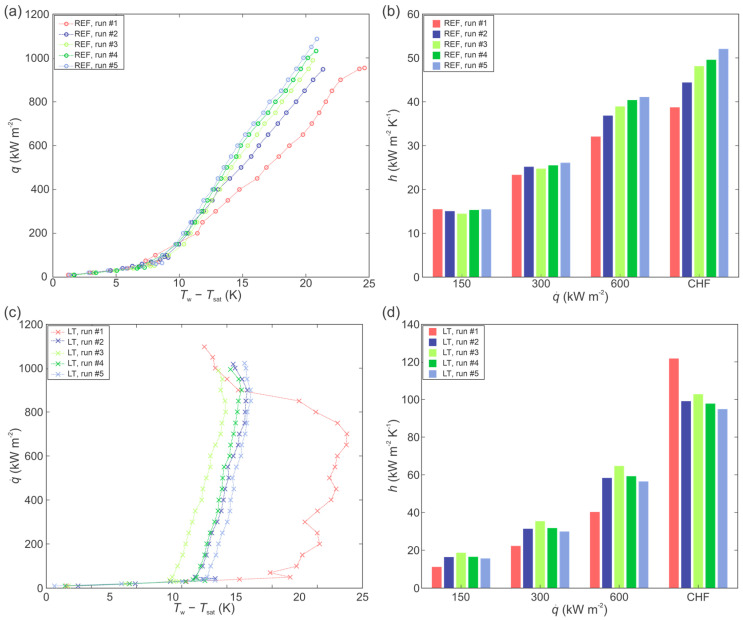
Boiling curves (**a**) and heat transfer coefficients (**b**) on the reference surface; boiling curves (**c**) and heat transfer coefficients (**d**) on the laser-textured surface.

To establish a baseline for experiments with nanofluids, the boiling performance of the bare copper surface and the laser textured surface was first evaluated utilizing twice-distilled water. The results are shown in Figure 3.

Figure 3a,c shows the boiling curves (i.e., heat flux as a function of surface superheat) recorded on the bare surface (REF) and the laser textured surface (LT), respectively. In both cases, the boiling curves were recorded until the incipience of the CHF. It is evident that the boiling curves on the REF surface are slightly shifted towards lower superheats after each experimental run. The same shifting of the boiling curve also occurs on the LT surface during the first two runs, but then boiling curves stabilize, with a slight reversal of the trend. This is also consistent with the wettability of the examined surfaces. The REF surface is transitioning from a hydrophilic state (before boiling) towards the hydrophobic state (after boiling), and the LT surface is transitioning from a super hydrophilic state to a hydrophobic state. This is in correlation with the previous research, where it was shown that the shift in the boiling curves ensues due to changes in surface morphology and chemistry [49,53]. The HTC of the REF and LT surfaces at selected heat fluxes are shown in Figure 3b,d, respectively.

The highest HTC values were recorded at the point of CHF incipience, where the LT surface provides a significant improvement compared to the untreated reference surface. Secondary boiling effects were detected on the LT surface, leading to a significant decrease in wall superheat temperature near the CHF, which in turn leads to the remarkable enhancement of the HTC. The reduction in surface superheats is caused by the higher density of active nucleation sites at high heat flux, when boiling also starts to take place on the peaks of the surface morphology instead of just within the cavities [46,55].

**Figure 4:**
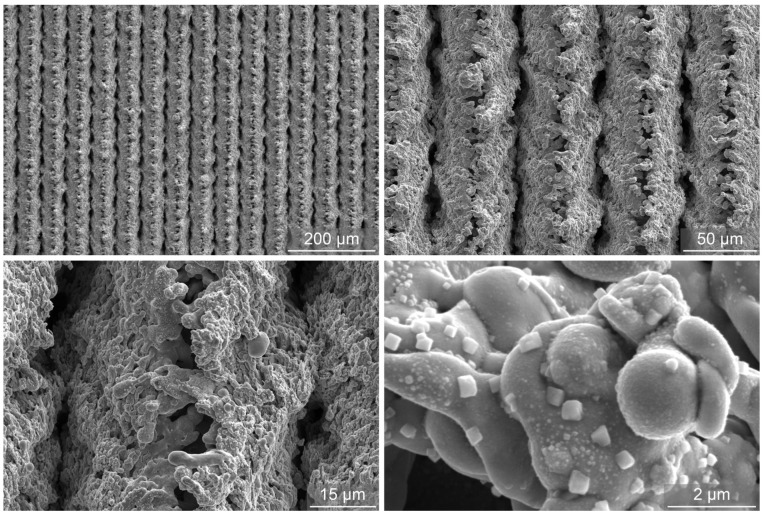
SEM images of the laser-textured sample LT after exposure to the boiling of water.

SEM images of the laser-textured sample (LT) after exposure to the boiling of water are shown in Figure 4. It is evident that the microtopography remains the same, but changes are present on the submicron scale, where the transition of oxide species from copper(II) oxide to copper(I) oxide is evident and perfectly matches previous observations [49,53]. In [49], the appearance of copper(I) oxide in the form of sub-micron sized cubes or (truncated) octahedra was shown to take place after CHF incipience as a result of low temperature annealing, resulting in a transition of needle-shaped copper(II) oxide in the reduced form.

### 3.2. The Effect of Concentration on Boiling of Nanofluid with Small and Large TiO_2_ Nanoparticles

**Figure 5:**
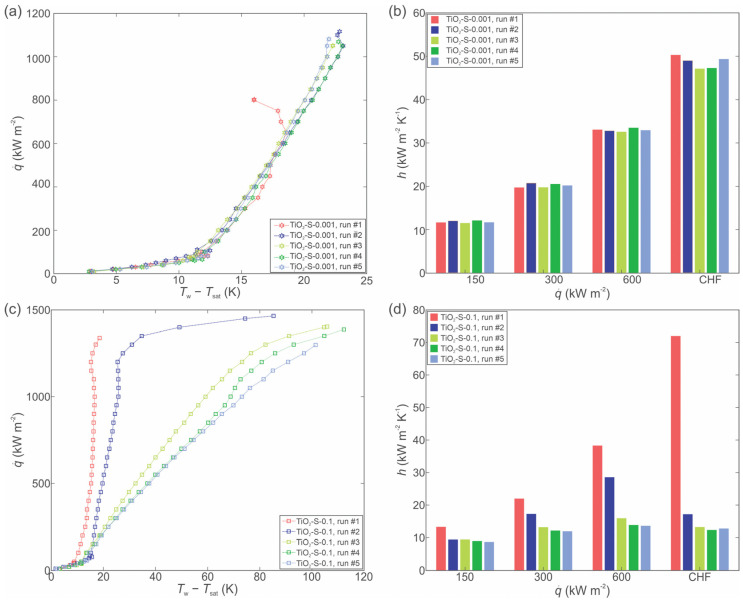
Boiling of TiO_2_-water nanofluid with small size nanoparticles at 0.001 wt.%: boiling curves (**a**) and heat transfer coefficients (**b**). Boiling of TiO_2_-water nanofluid with small size nanoparticles at 0.1 wt.%: boiling curves (**c**) and heat transfer coefficients (**d**).

The boiling of nanofluid with small (4–8 nm) TiO_2_ nanoparticles on the copper laser textured surface was evaluated at two different mass concentrations of 0.001 and 0.1 wt.%. The corresponding boiling curves and HTCs are shown in Figure 5a,b for 0.001 wt.% nanofluid, while the data for the 0.1 wt.% nanofluid is shown in Figure 5c,d. The boiling curves obtained using 0.001 wt.% nanofluid are very stable, while the curves for the 0.1 wt.% nanofluid are shifted towards higher superheat temperatures with each consecutive run. This shifting can be explained through a decrease in the active nucleation site density [33,35] due to the deposition of nanoparticles onto the surface. At 0.1 wt.%, many microcavities on the laser textured surface become filled with nanoparticles during the first experimental run, which leads to a decrease in active nucleation site density and an increase in surface superheat during the next boiling runs. With increasing the number of runs, the surface superheat increases to unacceptable levels for practical use and the boiling performance is deteriorated. The highest HTC value, recorded during the first run (approx. 1 h of boiling on the surface), was 79,4 kW m^−2^ K^−1^ near CHF incipience. After the 5th run, the HTC value was 83% lower at same heat flux. During the second boiling run, the high surface temperature prevented the CHF incipience from being recorded, as the setup was turned off due to safety reasons. The highest CHF of 1457 kW m^−2^ was recorded at 0.1 wt.%, which represents an enhancement of 35% compared to the highest recorded CHF at 0.001 wt.% (1082 kWm^−2^). The enhancement of CHF is attributed due to a reduction in static contact angle and an improvement in surface wettability.

**Figure 6:**
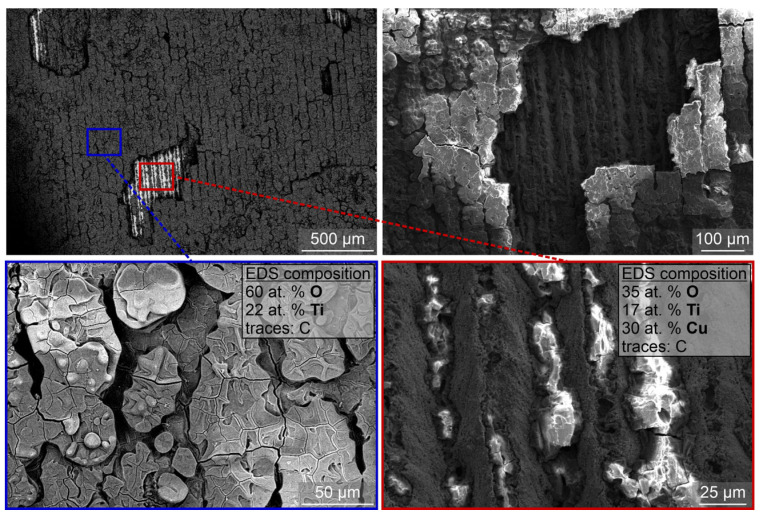
SEM images of the laser-textured sample LT after exposure to boiling 0.1 wt.% nanofluid with small nanoparticles (i.e., S-0.1).

SEM images of the laser-textured sample after exposure to boiling of 0.1 wt.% nanofluid with smaller nanoparticles are shown in Figure 6. It is observable that a thick nanoparticle deposit has formed on the surface, obscuring the shape of the laser-induced microstructure below. However, the deposited layer is not homogeneous and uniform, as evident from the missing patches, under which the laser-textured copper surface is visible. This was confirmed through EDS analysis, which detected a mixture of titanium and oxygen stemming from the TiO_2_ deposits on most of the surface, while a lower percentage of these two elements was detected, along with a notable percentage of copper on the flaked-off patch.

**Figure 7:**
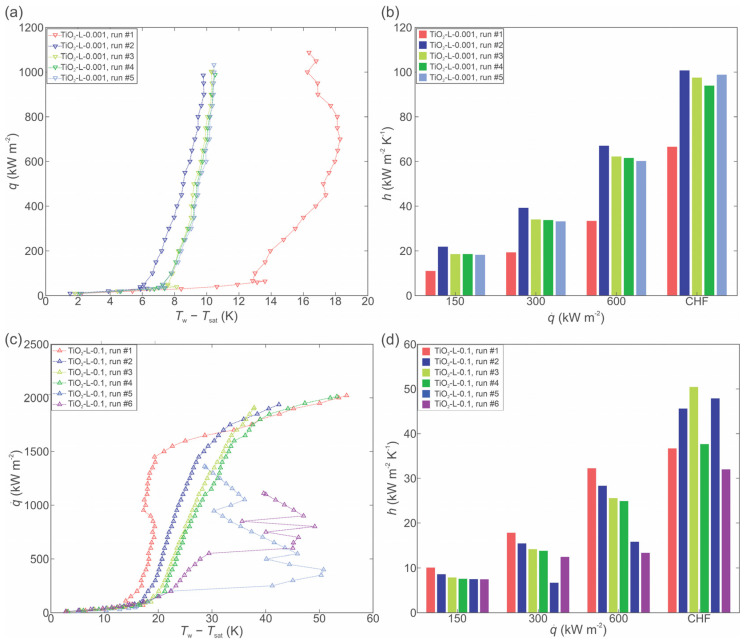
Boiling of TiO_2_-water nanofluid with large size nanoparticles at 0.001 wt.%: boiling curves (**a**) and heat transfer coefficients (**b**). Boiling of TiO_2_-water nanofluid with large size nanoparticles at 0.1 wt.%: boiling curves (**c**) and heat transfer coefficients (**d**).

The boiling of nanofluid with larger nanoparticles (490 nm) was performed at 0.001 and 0.1 wt.%. Boiling curves at 0.001 and 0.1 wt.% are shown in Figure 7a,c, respectively, while the HTCs are shown in Figure 7b,d, respectively.

**Figure 8:**
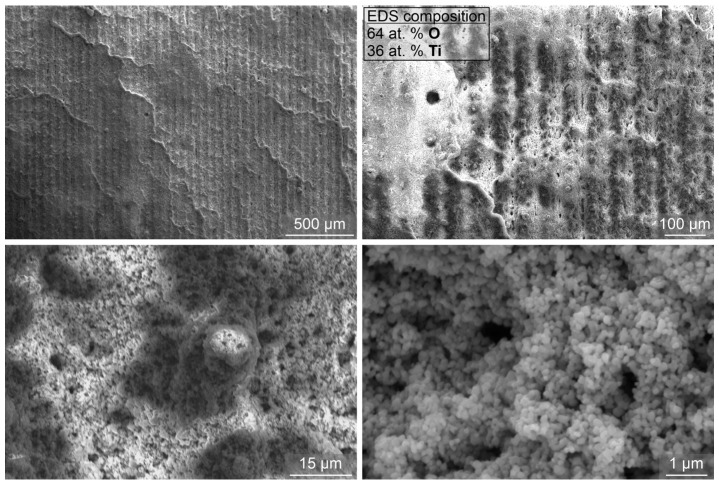
SEM images of the laser-textured sample LT after exposure to boiling 0.1 wt.% nanofluid with large nanoparticles (i.e., L-0.1).

At 0.001 wt.%, a significant shift in the boiling curves towards a lower surface superheat was observed, while at 0.1 wt.%, the boiling curves are shifted toward higher superheat values. The general observations match those made for the smaller nanoparticle size. However, it was observed that after prolonged boiling of highly concentrated nanofluid (0.1 wt.% for 4+ h), the boiling curves became very unstable (e.g., Figure 7c) due to the very thick deposited layer on the surface, which also flakes off locally. SEM images of the laser-textured sample after exposure to the boiling of 0.1 wt.% nanofluid with larger nanoparticles are shown in Figure 8. Here, the underlying laser-induced microstructure is evident, but the deposited layer is again heterogeneous. It is estimated that significant flaking of the deposited layer occurred during the boiling process and after removal of the sample due to the low adhesion strength. The EDS analysis confirmed a nearly perfect atomic ratio of oxygen to titanium (2:1) for the TiO_2_ deposit.

**Figure 9:**
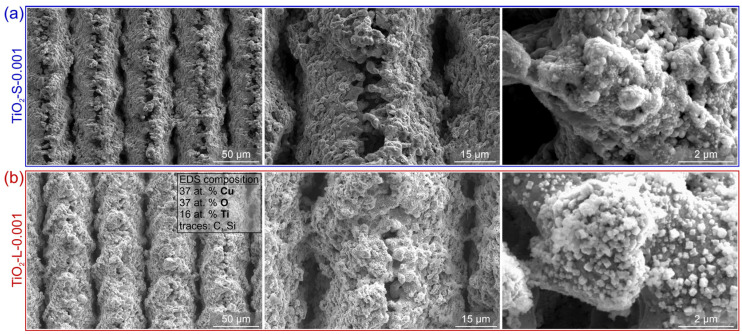
(**a**) SEM images of the laser-textured sample LT after exposure to boiling 0.001 wt.% nanofluid with small nanoparticles (i.e., S-0.001) and (**b**) after exposure to boiling 0.001 wt.% nanofluid with large nanoparticles (i.e., L-0.001).

Interestingly, the boiling performance increase detected with larger nanoparticles at 0.001 wt.% was not observed at the same concentration using the smaller nanoparticle size. It is likely that the small nanoparticles (two orders of magnitude smaller than the large nanoparticles used in this study) form a thin, compact deposited layer without additional nucleation sites (i.e., the HTC is not enhanced at comparable heat flux values), while the increased wettability is able to raise the CHF value. On the other hand, much larger nanoparticles (490 nm) form a more porous deposit, which offers additional cavities for bubble nucleation, thus enhancing the HTC, but the overall effect on increasing the surface wettability (and with that, the CHF value) is lower. A comparison of the SEM images taken after boiling of 0.001 wt.% nanofluid, with both small and large nanoparticle size, is shown in Figure 9. Only a thin layer of small nanoparticles remains on the laser-induced structured after boiling the 0.001 wt.% nanofluid and is only evident at higher magnifications. On the contrary, larger nanoparticles deposited at the same concentration are more clearly visible and seem to fill a part of the laser-induced surface channels.

The HTC is enhanced by 49% after 5 h of boiling at the concentration of 0.001 wt.%, and both the CHF and the HTC enhancements are stable. The CHF enhancement is more pronounced with larger nanoparticles at 0.1 wt.%, with the highest value reaching 2021 kW m^−2^, which represents an 86% enhancement over the highest CHF recorded for the 0.001 wt.% Enhancement of the CHF was recorded for all performed runs, which was attributed to better wettability characteristics of the surface exposed to the boiling of the concentrated nanofluid. Increased wettability compared to the results for the boiling of pure water on the LT surface was confirmed by the measurement of the static contact angle after boiling. The contact angle after boiling with the 0.1 wt.% nanofluid was 13.5°, and 29.8° for the 0.001 wt.% nanofluid.

**Figure 10:**
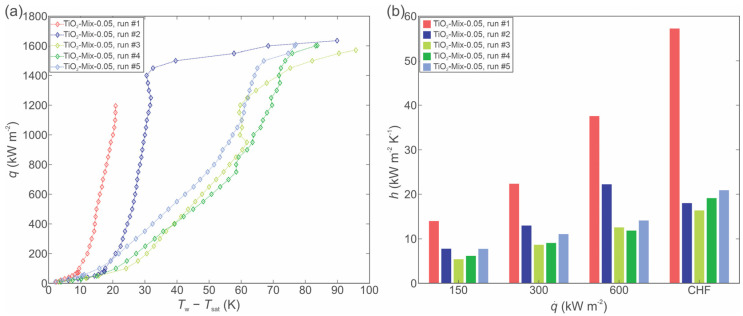
Boiling of TiO_2_-water nanofluid with 0.05 wt.% of small size and 0.05 wt.% large size nanoparticles, respectively: boiling curves (**a**) and heat transfer coefficients (**b**).

A further investigation was performed by mixing 0.05 wt.% of small and 0.05 wt.% of large size nanoparticles, respectively. The boiling curves and HTCs are shown in Figure 10.

The boiling curves are again shifted toward a higher surface superheat, which matches the behavior when 0.1 wt.% nanofluid was boiled with either small or large nanoparticles. After the first experimental run, the surface superheat increased significantly, which was likely caused by the presence of small size nanoparticles that filled the microcavities and surface channels, thus decreasing the active nucleation site density. As the latter decreases, so does the HTC. CHF was again not recorded after the first hour of boiling due to very high surface temperatures and the danger of damage to the setup, but the achieved maximal heat flux values were notably lower than for the 0.1 wt.% nanofluid with large particles, but higher than for the same concentration of nanofluid with small nanoparticles.

**Figure 11:**
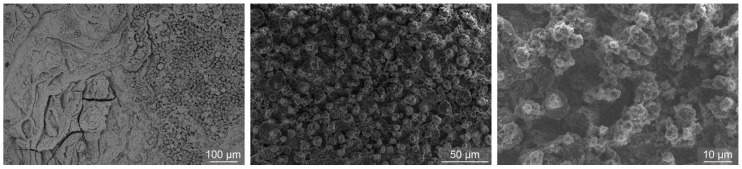
SEM images of the laser-textured sample LT after exposure to boiling of nanofluid with a mixture of 0.05 wt.% of small nanoparticles and 0.05 wt.% of large nanoparticles (i.e., MIX-0.05).

SEM images of the surface after exposure to the boiling of nanofluid with mixed particle size (i.e., MIX-0.05) are shown in Figure 11.

It is noticeable that the surface is rather uniformly covered with a deposited layer, and the laser-induced structures are not visible. However, the deposit is again inhomogeneous.

### 3.3. Contact Angle Measurments

**Figure 12:**
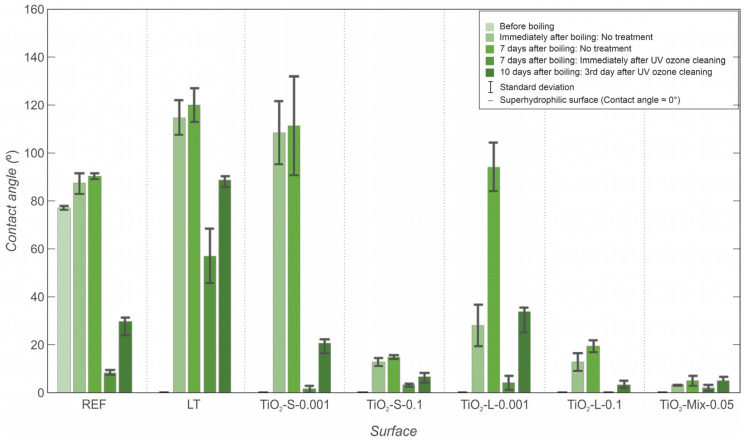
Static contact angle measurements on all surfaces before boiling, after boiling, and after storage.

The static contact angle of water was measured on each surface to determine its wettability and help explain the observed boiling behaviors, especially in terms of the effect of nanoparticle deposition onto the surface during the experiments. The recorded values, obtained through measurements at different points in time, as previously explained in Section 2.1, are depicted and compared in Figure 12.

Laser textured surfaces were initially superhydrophilic immediately after processing, and the contact angle of the bare copper surface was 82° before the boiling experiments. After the boiling experiments with water on the laser textured surface and the reference surface, the contact angles increased, as was shown in previous studies [51]. After the boiling experiments, the wettability of the surfaces changed due to the deposition of nanoparticles. The increase in the contact angles of surfaces is slightly higher after boiling with low-concentration nanofluids than for the contact angles measured after boiling with high-concentration nanofluids. This could be due to the higher deposition rate at higher concentrations, which causes a thicker layer of deposited TiO_2_ nanoparticles on the surface. TiO_2_ nanoparticles are (super)hydrophilic, and a thick layer of them on the surface leads to the surface staying in a hydrophilic state. At lower concentrations and for the small-sized nanoparticles, the deposited layer on the surface is thinner. On the other hand, the larger nanoparticles form more porous deposits, resulting in lower contact angles compared to those for the smaller nanoparticles used in this study.

Additionally, all surfaces were exposed to ambient conditions for several days after the boiling experiments were finished and then cleaned with a UV/ozone cleaner to remove volatile organic compounds (VOC) and other carbon-based impurities. Contact angle measurements were performed before and immediately after cleaning with the UV/ozone cleaner. Afterward, the surfaces were again exposed to ambient air for three days, and the contact angles were measured again. The results of the measurements show the contact angles of all surfaces gradually increased over time, which was observed in many previous studies [56,57], and this was confirmed by previous research [58,59]. The change in the wettability is attributed to the adsorption of hydrophobic contaminants from the air. The adsorption of polymeric organosilicon compounds was recently found to be important as the most probable reason for the wettability transition of such samples in the laboratory environment [60]. The air-exposed surfaces were then cleaned with a UV/ozone cleaner for 30 min. This removes most typical contaminants, such as oils and greases, and other contamination adsorbed during prolonged exposure to air [61]. The wettability of all surfaces increased dramatically after the UV/ozone cleaning, but after exposing them to the ambient air conditions for a further 3 days, the wettability again decreased.

### 3.4. Comparison of the Overall Boiling Performance

It was observed that regardless of the size of the nanoparticle, an increase in concentration had the same effect on the CHF and HTC, which agrees with the findings of previous research [33,35,62,63,64]. The research groups in this field concluded that an increase in nanoparticle concentration leads to the creation of a fouling layer on the surface, which deteriorates HTC due to a decrease in active nucleation site density. In our case, the laser-induced microcavities were filled with nanoparticles, leading to a decrease in contact angle and a reduction in the number of cavities suitable for bubble nucleation [65,66]. Additionally, the adhesion energy, which is defined as the horizontal component of the surface tension force acting against the bubble growth, significantly increased with nanoparticle deposition. This caused the bubble departure frequency to decrease and prolonged their growth times. On top of that, the fluid was wetting the entire deposited layer, which lead to deterioration of the active nucleation sites on the surface [67]. On the other hand, the layer formed on the surface improved the wettability, capillary wicking action, and constitution of inflow liquid inside the fouling layer [62,63,68], which effectively increased the achievable heat flux values without CHF incipience.

**Figure 13:**
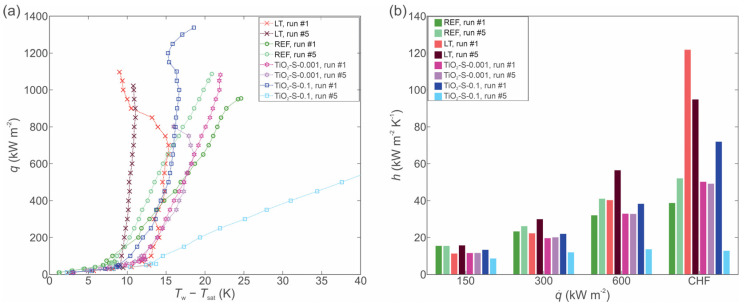
Comparison of boiling characteristics of TiO_2_-water nanofluids with small size nanoparticles with the performance of pure water on reference and LT surface (1st and 5th run): boiling curves (**a**) and heat transfer coefficients (**b**).

A comparison of the boiling performance of nanofluids with small nanoparticles with the performance of twice-distilled water on the reference and LT surface is shown in Figure 13. Nanofluids at both concentrations deteriorated the heat transfer parameters, in comparison with the boiling of pure water, on the LT surface. Additionally, at the low concentration, the boiling performance also decreased compared to the boiling performance of water on the untreated reference surface. The highest deterioration in HTC recorded at CHF was measured to be 60% at the low and 89.5% at the high nanoparticle concentration compared to the HTC recorded on the LT surface with the base fluid (water) at CHF.

**Figure 14:**
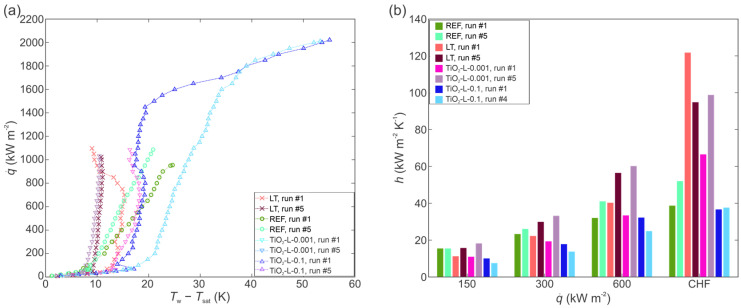
Comparison of boiling characteristics of TiO_2_-water nanofluids-small size NPs with base fluid on the reference and LT surface (1st and 5th run): boiling curves (**a**) and HTCs (**b**).

Furthermore, Figure 14 shows a comparison of the boiling performance of nanofluids with the performance of twice-distilled water on the reference and LT surface. Here, a deterioration of the HTC when using the nanofluid instead of pure water is also recorded.

**Figure 15:**
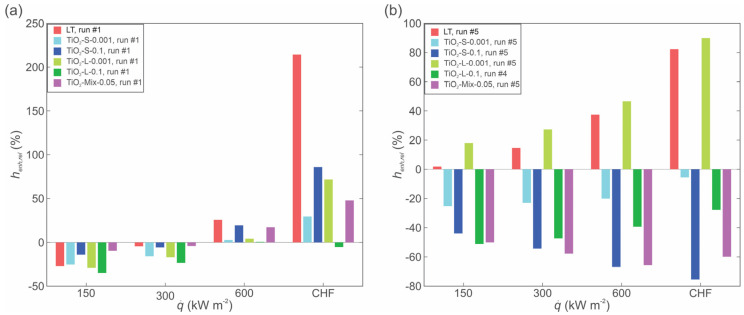
Comparison of HTC for different boiling media (pure water or nanofluids) relative to the performance of the untreated reference surface during the 1st run (**a**) and during the 5th run (**b**).

Figure 15 shows a comparison of the performance of the laser-textured surface, when used either with water (denoted as LT), or with nanofluids, relative to the boiling performance of pure water on the untreated reference surface (REF). At low and medium heat flux values, the reference surface provides superior performance during the first experimental run and only at high heat fluxes, the laser-texturing and nanoparticle deposition enhance the HTC (Figure 15a). However, after 5 experimental runs were performed on each surface, the performance of the laser-textured surface used with pure water or 0.001 wt.% large nanoparticle nanofluid significantly exceeds the performance of the untreated surfaces. This is mainly attributed to the increased number of potential active nucleation sites on both surfaces, as shown in the SEM images and discussed previously. On the other hand, the deterioration of HTC is exacerbated on other surfaces, with its values being up to 75% lower compared to those of pure water boiling on the untreated surface. The main cause for the deterioration of boiling performance is the growth of a thick nanoparticle deposit layer, which raises the surface temperature due to increased thermal resistance of the deposited titanium dioxide layer. The obtained results are in accordance with the findings of other authors for large nanoparticles and mixed sizes of nanoparticles [69], and also for nanofluids with small nanoparticle diameters [70].

Significant enhancement of the CHF was not observed at 0.001 wt.% compared to the CHF for pure water on either the laser-textured or untreated surface, while the highly concentrated nanofluid evidently caused thicker deposits, resulting in significant increases in the CHF value. Enhanced CHF can be mainly be attributed to the increased wettability [69,71] of the surfaces and their porosity; this also agrees with the contact angle measurements after boiling, shown in Figure 12.

**Figure 16:**
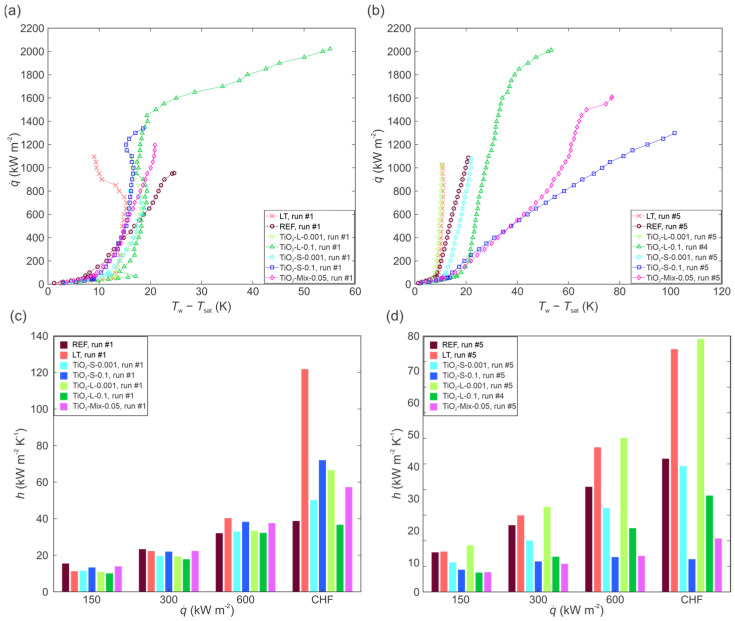
Summary of boiling performance for all tested cases: boiling curves during the first run (**a**) and during the last run (**b**) on each surface; HTC values during the first run (**c**) and during the last run (**d**) on each surface.

Finally, a comparison of all boiling curves for the first and last experimental run is made in Figure 16a,b, respectively, to elucidate the effect of both nanoparticle concentration and size on boiling performance. Additionally, the corresponding plots comparing HTC values at selected heat fluxes are shown in Figure 16c,d.

During the first experimental run, an enhancement of boiling performance in terms of increased HTC at medium and high heat flux values was observed for the laser-textured surface tested with pure water, for both mixed nanoparticle size nanofluid and for both low-concentration nanofluids (0.001 wt.%). A noticeable CHF increase was obtained with highly concentrated nanofluids (0.1 wt.%), but high surface superheat values were already recorded for the nanofluid with large nanoparticles, hinting at thicker deposits and problems during future testing. It can be concluded that large nanoparticles agglomerate much faster, which improves the deposition rate, leading to a reduction in the number of active nucleation sites. This observation is not in accordance with previous research, which concluded that if the size of nanoparticles is much smaller than the roughness of the surface, an enhancement of boiling performance will be achieved. While even the large nanoparticles are approximately two orders of magnitude smaller than the laser-induced roughness, a deterioration in boiling performance was observed, matching previous reports for larger ratios of nanoparticle size to surface roughness [70,72]. Here, we report that regardless of the sizes of nanoparticles, the HTC of nanofluids at the higher concentration on laser-textured surface decreases compared to the boiling of water on the same surface. The last experimental run on each surface revealed that highly concentrated nanofluids provided a notable CHF enhancement, but universally at the expense of notable surface superheat increase, which reached 100 K for nanofluid containing 0.1 wt.% of small nanoparticles. Overall, CHF enhancement due to the boiling of nanofluid on laser-textured surfaces was not observed at the low concentration.

## 4. Conclusions

The pool boiling performance of TiO_2_-water nanofluids with two different sizes of nanoparticles (4–8 nm and 490 nm) at two different concentrations (0.001 wt.% and 0.1 wt.%) was investigated on laser-textured copper surfaces. The following conclusions were made based on the results of the performed experiments:

1. The boiling of nanofluids on an already enhanced (i.e., laser-textured) surface failed to provide a notable (additional) enhancement of the heat transfer coefficient.
2. At a low nanoparticle concentration, the influence of nanofluid on boiling performance is minimal, with heat transfer coefficient and CHF values comparable to those obtained using pure water on both the untreated and laser-textured surface.
3. The boiling of a nanofluid with a high nanoparticle concentration resulted in significant deposition of nanoparticles onto the boiling surface and CHF enhancement up to 2021 kW m^−2^, representing double the value obtained on the untreated reference surface using water. However, very high surface superheat values (up to 100 K) were recorded, suggesting poor practical applicability.
4. The decrease in heat transfer performance due to the boiling of nanofluids on laser-textured surfaces can be explained through the deposition of nanoparticles into the laser-induced grooves and microcavities present on the surface, which decreased the number of active nucleation sites. Furthermore, thicker nanoparticle deposits resulted in added thermal resistance. While the surface porosity granted a notable delay in CHF incipience due to enhanced liquid replenishment, the surface superheat was massively increased.

In summary, the use of nanofluids on surfaces previously functionalized with surface treatment methods (e.g., laser texturing) was generally proved to be unable to provide an additional viable heat transfer performance enhancement.

## Figures and Tables

**Table 1:** The characteristics of the performed experiments.

Name	Boiling Fluid	Surface	Size of Nanoparticles	Concentration (wt.%)
REF	water	Bare copper	/	/
LT	water	laser textured copper	/	/
TiO_2_-S-0.001	TiO_2_-water		small (4–8 nm)	0.001
TiO_2_-S-0.1	TiO_2_-water		large (490 nm)	0.1
TiO_2_-L-0.001	TiO_2_-water		small (4–8 nm)	0.001
TiO_2_-L-0.1	TiO_2_-water		large (490 nm)	0.1
TiO_2_-Mix-0.05	TiO_2_-water		small (4–8 nm) + large (490 nm)	0.05 small and 0.05 large

## Data Availability

All data is available from the corresponding author upon request.

## References

[1] Dhir V.K. (1991). Nucleate and Transition Boiling Heat Transfer Under Pool and External Flow Conditions. Int. J. Heat Fluid Flow.

[2] Liang G., Mudawar I. (2017). Review of spray cooling—Part 1: Single-phase and nucleate boiling regimes, and critical heat flux. Int. J. Heat Mass Transf..

[3] Mori S., Utaka Y. (2017). Critical heat flux enhancement by surface modification in a saturated pool boiling: A review. Int. J. Heat Mass Transf..

[4] Zhang C., Yu F., Li X., Chen Y. (2019). Gravity–capillary evaporation regimes in microgrooves. AIChE J..

[5] Zhang C., Chen Y., Wu R., Shi M. (2011). Flow boiling in constructal tree-shaped minichannel network. Int. J. Heat Mass Transf..

[6] Mudawar I. (2013). Recent advances in high-flux, two-phase thermal management. J. Therm. Sci. Eng. Appl..

[7] Nukiyama S. (1966). The maximum and minimum values of the heat Q transmitted from metal to boiling water under atmospheric pressure. Int. J. Heat Mass Transf..

[8] Lahey R.T. (1992). Boiling Heat Transfer: Modern Developments and Advances.

[9] Hewitt G.F., Tien C.L. (1997). Series in Chemical and Mechanical Engineering Tong and Tang, Boiling Heat Transfer and Two-Phase Flow.

[10] Malavasi I., Teodori E., Moita A.S., Moreira A.L.N., Marengo M. (2018). Wettability Effect on Pool Boiling: A Review. Encyclopedia of Two-Phase Heat Transfer and Flow III.

[11] Liang G., Mudawar I. (2019). Review of pool boiling enhancement by surface modification. Int. J. Heat Mass Transf..

[12] Pioro I.L., Rohsenow W., Doerffer S.S. (2004). Nucleate pool-boiling heat transfer. I: Review of parametric effects of boiling surface. Int. J. Heat Mass Transf..

[13] Može M., Vajc V., Zupančič M., Golobič I. (2021). Hydrophilic and hydrophobic nanostructured copper surfaces for efficient pool boiling heat transfer with water, water/butanol mixtures and Novec 649. Nanomaterials.

[14] Zupančič M., Može M., Gregorčič P., Golobič I. (2017). Nanosecond laser texturing of uniformly and non-uniformly wettable micro structured metal surfaces for enhanced boiling heat transfer. Appl. Surf. Sci..

[15] Chu K.H., Enright R., Wang E.N. (2012). Structured surfaces for enhanced pool boiling heat transfer. Appl. Phys. Lett..

[16] Ji X., Xu J., Zhao Z., Yang W. (2013). Pool boiling heat transfer on uniform and non-uniform porous coating surfaces. Exp. Therm. Fluid Sci..

[17] Li J.Q., Mou L.W., Zhang J.Y., Zhang Y.H., Fan L.W. (2018). Enhanced pool boiling heat transfer during quenching of water on superhydrophilic porous surfaces: Effects of the surface wickability. Int. J. Heat Mass Transf..

[18] Lee C.Y., Zhang B.J., Kim K.J. (2012). Morphological change of plain and nano-porous surfaces during boiling and its effect on nucleate pool boiling heat transfer. Exp. Therm. Fluid Sci..

[19] Shi J., Jia X., Feng D., Chen Z., Dang C. (2020). Wettability effect on pool boiling heat transfer using a multiscale copper foam surface. Int. J. Heat Mass Transf..

[20] Li S., Furberg R., Toprak M.S., Palm B., Muhammed M. (2008). Nature-inspired boiling enhancement by novel nanostructured macroporous surfaces. Adv. Funct. Mater..

[21] Lee S.W., Park S.D., Bang I.C. (2012). Critical heat flux for CuO nanofluid fabricated by pulsed laser ablation differentiating deposition characteristics. Int. J. Heat Mass Transf..

[22] Ahn H.S., Sathyamurthi V., Banerjee D. (2009). Pool boiling experiments on a nano-structured surface. IEEE Trans. Compon. Packag. Technol..

[23] Gupta S.K., Misra R.D. (2018). Experimental study of pool boiling heat transfer on copper surfaces with Cu-Al_2_O_3_ nanocomposite coatings. Int. Commun. Heat Mass Transf..

[24] O’Connor J.P., You S.M., Chang J.Y. (1996). Gas-Saturated Pool Boiling Heat Transfer from Smooth and Microporous Surfaces in FC-72. ASME J. Heat Transf..

[25] Takata Y., Hidaka S., Masuda M., Ito T. (2003). Pool boiling on a superhydrophilic surface. Int. J. Energy Res..

[26] O’Hanley H., Coyle C., Buongiorno J., McKrell T., Hu L.W., Rubner M., Cohen R. (2013). Separate effects of surface roughness, wettability, and porosity on the boiling critical heat flux. Appl. Phys. Lett..

[27] Kim S.J., Bang I.C., Buongiorno J., Hu L.W. (2006). Effects of nanoparticle deposition on surface wettability influencing boiling heat transfer in nanofluids. Appl. Phys. Lett..

[28] Jo H., Ahn H.S., Kang S., Kim M.H. (2011). A study of nucleate boiling heat transfer on hydrophilic, hydrophobic and heterogeneous wetting surfaces. Int. J. Heat Mass Transf..

[29] Phan H.T., Caney N., Marty P., Colasson S., Gavillet J. (2009). Surface wettability control by nanocoating: The effects on pool boiling heat transfer and nucleation mechanism. Int. J. Heat Mass Transf..

[30] Barber J., Brutin D., Tadrist L. (2011). A review on boiling heat transfer enhancement with nanofluids. Nanoscale Res. Lett..

[31] Fang X., Chen Y., Zhang H., Chen W., Dong A., Wang R. (2016). Heat transfer and critical heat flux of nanofluid boiling: A comprehensive review. Renew. Sustain. Energy Rev..

[32] Gerardi C., Buongiorno J., Hu L.-W., McKrell T. (2011). Infrared thermometry study of nanofluid pool boiling phenomena. Nanoscale Res. Lett..

[33] Vafaei S., Borca-Tasciuc T. (2014). Role of nanoparticles on nanofluid boiling phenomenon: Nanoparticle deposition. Chem. Eng. Res. Des..

[34] Murshed S.M.S., Nieto De Castro C.A., Loureno M.J.V., Lopes M.L.M., Santos F.J.V. (2011). A review of boiling and convective heat transfer with nanofluids. Renew. Sustain. Energy Rev..

[35] Khan A., Ali H.M. (2019). A comprehensive review pool boiling using nanofluids. Therm. Sci..

[36] Dadhich M., Prajapati O.S. (2019). A brief review on factors affecting flow and pool boiling. Renew. Sustain. Energy Rev..

[37] Manetti L.L., Stephen M.T., Beck P.A., Cardoso E.M. (2017). Evaluation of the heat transfer enhancement during pool boiling using low concentrations of Al2O3-water based nanofluid. Exp. Therm. Fluid Sci..

[38] Ahmed O., Hamed M.S. (2012). Experimental investigation of the effect of particle deposition on pool boiling of nanofluids. Int. J. Heat Mass Transf..

[39] Huang C.K., Lee C.W., Wang C.K. (2011). Boiling enhancement by TiO_2_ nanoparticle deposition. Int. J. Heat Mass Transf..

[40] Kiyomura I.S., Manetti L.L., da Cunha A.P., Ribatski G., Cardoso E.M. (2017). An analysis of the effects of nanoparticles deposition on characteristics of the heating surface and ON pool boiling of water. Int. J. Heat Mass Transf..

[41] Salimpour M.R., Abdollahi A., Afrand M. (2017). An experimental study on deposited surfaces due to nanofluid pool boiling: Comparison between rough and smooth surfaces. Exp. Therm. Fluid Sci..

[42] Taylor R.A., Phelan P.E. (2009). Pool boiling of nanofluids: Comprehensive review of existing data and limited new data. Int. J. Heat Mass Transf..

[43] Park S.D., Moon S.B., Bang I.C. (2014). Effects of thickness of boiling-induced nanoparticle deposition on the saturation of critical heat flux enhancement. Int. J. Heat Mass Transf..

[44] Yim K., Lee J., Naccarato B., Kim K.J. (2019). Surface wettability effect on nucleate pool boiling heat transfer with titanium oxide (TiO2) coated heating surface. Int. J. Heat Mass Transf..

[45] Može M., Senegačnik M., Gregorčič P., Hočevar M., Zupančič M., Golobič I. (2020). Laser-Engineered Microcavity Surfaces with a Nanoscale Superhydrophobic Coating for Extreme Boiling Performance. ACS Appl. Mater. Interfaces.

[46] Kruse C.M., Anderson T., Wilson C., Zuhlke C., Alexander D., Gogos G., Ndao S. (2015). Enhanced pool-boiling heat transfer and critical heat flux on femtosecond laser processed stainless steel surfaces. Int. J. Heat Mass Transf..

[47] Zakšek P., Zupančič M., Gregorčič P., Golobič I. (2020). Investigation of Nucleate Pool Boiling of Saturated Pure Liquids and Ethanol-Water Mixtures on Smooth and Laser-Textured Surfaces. Nanoscale Microscale Thermophys. Eng..

[48] Serdyukov V., Starinskiy S., Malakhov I., Safonov A., Surtaev A. (2021). Laser texturing of silicon surface to enhance nucleate pool boiling heat transfer. Appl. Therm. Eng..

[49] Može M., Zupančič M., Hočevar M., Golobič I., Gregorčič P. (2019). Surface chemistry and morphology transition induced by critical heat flux incipience on laser-textured copper surfaces. Appl. Surf. Sci..

[50] Karthikeyan A., Coulombe S., Kietzig A.M. (2018). Boiling heat transfer enhancement with stable nanofluids and laser textured copper surfaces. Int. J. Heat Mass Transf..

[51] Freitas E., Pontes P., Cautela R., Bahadur V., Miranda J., Ribeiro A.P.C., Souza R.R., Oliveira J.D., Copetti J.B., Lima R. (2021). Article pool boiling of nanofluids on biphilic surfaces: An experimental and numerical study. Nanomaterials.

[52] Pontes P., Freitas E., Fernandes D., Teixeira A., Ferreira R., Bellmann S., Cautela R., Moita A.S., Bahadur V., Miranda J. (2021). Pool boiling of nanofluids on biphilic surfaces. Proceedings of the 8th European Thermal Sciences Conference (EUROTHERMAL 2021).

[53] Može M., Vajc V., Zupančič M., Šulc R., Golobič I. (2021). Pool boiling performance of water and self-rewetting fluids on hybrid functionalized aluminum surfaces. Processes.

[54] Može M., Zupančič M., Golobič I. (2020). Investigation of the scatter in reported pool boiling CHF measurements including analysis of heat flux and measurement uncertainty evaluation methodology. Appl. Therm. Eng..

[55] Može M., Zupančič M., Golobič I. (2020). Pattern geometry optimization on superbiphilic aluminum surfaces for enhanced pool boiling heat transfer. Int. J. Heat Mass Transf..

[56] Gregorčič P., Conradi M., Hribar L., Hočevar M. (2018). Long-term influence of laser-processing parameters on (Super)hydrophobicity development and stability of stainless-steel surfaces. Materials.

[57] Trdan U., Hočevar M., Gregorčič P. (2017). Transition from superhydrophilic to superhydrophobic state of laser textured stainless steel surface and its effect on corrosion resistance. Corros. Sci..

[58] Ta D.V., Dunn A., Wasley T.J., Kay R.W., Stringer J., Smith P.J., Connaughton C., Shephard J.D. (2015). Nanosecond laser textured superhydrophobic metallic surfaces and their chemical sensing applications. Appl. Surf. Sci..

[59] Long J., Zhong M., Fan P., Gong D., Zhang H. (2015). Wettability conversion of ultrafast laser structured copper surface. J. Laser Appl..

[60] Gregorčič P. (2020). Comment on “Bioinspired Reversible Switch between Underwater Superoleophobicity/Superaerophobicity and Oleophilicity/Aerophilicity and Improved Antireflective Property on the Nanosecond Laser-Ablated Superhydrophobic Titanium Surfaces”. ACS Appl. Mater. Interfaces.

[61] Vig J.R., Bus J.W.L. (1976). UV/Ozone Cleaning of Surfaces. IEEE Trans. Parts Hybrids Packag..

[62] Vafaei S. (2015). Nanofluid pool boiling heat transfer phenomenon. Powder Technol..

[63] Sarafraz M.M., Hormozi F., Peyghambarzadeh S.M. (2016). Pool boiling heat transfer to aqueous alumina nano-fluids on the plain and concentric circular micro-structured (CCM) surfaces. Exp. Therm. Fluid Sci..

[64] Coursey J.S., Kim J. (2008). Nanofluid boiling: The effect of surface wettability. Int. J. Heat Fluid Flow.

[65] Park K.J., Jung D., Shim S.E. (2009). Nucleate boiling heat transfer in aqueous solutions with carbon nanotubes up to critical heat fluxes. Int. J. Multiph. Flow.

[66] Kathiravan R., Kumar R., Gupta A., Chandra R. (2009). Characterization and pool boiling heat transfer studies of nanofluids. J. Heat Transf..

[67] Ciloglu D., Bolukbasi A. (2015). A comprehensive review on pool boiling of nanofluids. Appl. Therm. Eng..

[68] Kim H.D., Kim M.H. CHF Enhancement in Pool Boiling of Nanofluid: Effect of Nanoparticle-Coating on Heating Surface. Proceedings of the KNS spring meeting.

[69] Suriyawong A., Wongwises S. (2010). Nucleate pool boiling heat transfer characteristics of TiO_2_-water nanofluids at very low concentrations. Exp. Therm. Fluid Sci..

[70] Hu Y., Li H., He Y., Liu Z., Zhao Y. (2017). Effect of nanoparticle size and concentration on boiling performance of SiO_2_ nanofluid. Int. J. Heat Mass Transf..

[71] Soltani S., Etemad S.G., Thibault J. (2009). Pool boiling heat transfer performance of Newtonian nanofluids. Heat Mass Transf. Stoffuebertragung.

[72] Das S.K., Prakash Narayan G., Baby A.K. (2008). Survey on nucleate pool boiling of nanofluids: The effect of particle size relative to roughness. J. Nanoparticle Res..

